# Subjective and objective vestibular changes that occur following paediatric cochlear implantation: systematic review and meta-analysis

**DOI:** 10.1186/s40463-019-0341-z

**Published:** 2019-05-22

**Authors:** Michael Yong, Emily Young, Jane Lea, Hannah Foggin, Erica Zaia, Frederick K. Kozak, Brian D. Westerberg

**Affiliations:** BC Rotary Hearing and Balance Centre, St. Paul’s Hospital, 1081 Burrard St, Vancouver, BC V6Z 1Y6 Canada

## Abstract

**Objective:**

Cochlear implantation can result in post-operative vestibular dysfunction of unknown clinical significance. The objective of this study was to characterize the presence, magnitude, and clinical significance of vestibular dysfunction that occurs after pediatric cochlear implantation.

**Data sources:**

The databases Embase, Medline (OvidSP), and PubMed were used. Only articles published in English were included. Grey literature and unpublished sources were also reviewed.

**Study selection:**

Articles published from 1980 until the present which documented pre-operative and post-operative vestibular testing on children under the age of 18 were used.

**Data extraction:**

Parameters that were assessed included number of patients, pre- and post-operative vestibular-evoked myogenic potentials (VEMPs), head impulse testing (HIT), calorics, and posturography, timing of pre- and post-operative testing, symptomatology, and other demographic data such as etiology of the hearing loss.

**Data synthesis:**

Ten articles were included. Relative risk values evaluating the effect of cochlear implantation on vestibular function were calculated for VEMPs and caloric testing due to the availability of published data. I^2^ values were calculated and 95% confidence intervals were reported. Separate analyses were conducted for each individual study and a pooled analysis was conducted to yield an overall relative risk. Assessment on risk of bias in individual studies and overall was performed.

**Conclusion:**

Pediatric cochlear implantation is associated with a statistically significant decrease in VEMP responses post-operatively (RR 1.8, *p* < 0.001, I^2^ 91.86, 95%CI 1.57–2.02). Similar results are not seen in caloric testing. Insufficient data is available for analysis of HIT and posturography. Further studies are necessary to determine the effect of cochlear implantation on objective vestibular measures post-operatively and whether any changes seen are clinically relevant in this population.

## Introduction

Cochlear implantation in the pediatric population has become a routine procedure for children with bilateral severe to profound sensorineural hearing loss [1]. The past decade has seen a rapid increase in the number of implants being performed due to cost-effectiveness studies and the improved quality of life enjoyed by implant recipients [2, 3]. However, there have been recent efforts aimed at further characterizing the potential side effects of cochlear implantation in children, specifically with respect to resultant vestibular dysfunction. There has been a growing trend towards bilateral simultaneous implantation in children due to recent studies showing an increased ability to preserve normal cortical preference and development, improved sound localization contributing to enhanced preverbal communication and language development, and greater cost-effectiveness [1, 4–6]. The impact that bilateral implantation has on vestibular and balance function in children has been studied but methodologies and results are variable, making it difficult to draw general conclusions from this research.

The vestibular ramifications of implantation in the adult population have been documented [7–9]. Cochlear implantation has the potential to cause a decrease in vestibular function in adults measured by objective testing such as vestibular evoked myogenic potentials (VEMPs), calorics, and head impulse testing (HIT) [8, 9]. In addition, a recent meta-analysis of vestibular function after cochlear implant surgery reported a significant negative effect on caloric and VEMP testing in adults, although the overall patient-reported symptomatic manifestations of these objective findings were found to be insignificant [10].

Documentation of pre- and post-operative vestibular function surrounding pediatric cochlear implantation are limited and perhaps reflective of the fact that many centres do not routinely perform such testing. This may be due to several reasons such as cost, unclear benefit, difficulty in testing very young children, and lack of available equipment. As such, no prior systematic reviews or meta-analyses are available. While pediatric cochlear implantation has proven to be a relatively safe procedure with few severe long-term complications, those related to the vestibular system have been reported [11, 12]. Data from the literature to date has shown that vestibular end-organ dysfunction can be a risk factor for implant failure in the pediatric population and that there may be value in identifying children with vestibular impairment after surgery to avoid this complication [13]. In addition, recent research has shown that cochlear implantation has the potential to affect vestibular function through direct current spread from the cochlea to the vestibular system [14].

The now commonplace nature of this procedure, including bilateral implantation, and the potential long-term ramifications of undetected vestibular damage highlights a need for systematic analysis of the current literature in order to develop evidence-informed decisions regarding implantation. In turn, this may further elucidate potential contraindications to surgery or highlight the need for earlier vestibular rehabilitation interventions following implantation in young patients.

### Objectives

The aim of this systematic review was to evaluate the vestibular side-effects of cochlear implantation in the pediatric population through examination of objective and subjective vestibular parameters. The review sought to answer the following questions:

1. Is there a significant decrease in objective vestibular testing responses in the implanted ear after cochlear implantation?
2. Are there any changes in subjective vestibular symptoms post-operatively that suggest persistent long-term clinical impairment?

According to the PICOS approach, the population of interest included pediatric patients under the age of 18 undergoing cochlear implantation who have had pre-operative and post-operative vestibular testing. Cochlear implantation represented the intervention, and the comparison or control was the pre-operative vestibular status of the patient prior to surgery. The outcome under investigation was the objective and subjective vestibular status of patients after surgery. The review was not limited to any specific study design criterium.

## Methods

Ethics exemption was obtained from the University of British Columbia Research Ethics Board. The Preferred Reporting Items for Systematic Reviews and Meta-analysis (PRISMA) statement was followed during the review.

### Protocol

Methods of analysis and inclusion/exclusion criteria were specified in advance and documented in a separate protocol.

### Study inclusion criteria

Prospective or retrospective cohort studies reporting both pre- and post-operative vestibular testing of pediatric patients under the age of 18 at the time of cochlear implantation were included. Studies including children who underwent either unilateral or bilateral cochlear implantation (simultaneous or sequential) were included. Studies that included adults as well as children were also included, providing individual relevant data was available for the pediatric patients. Children with additional comorbidities were included, even those with conditions that predispose to vestibular dysfunction, as these form an important cohort of patients currently undergoing cochlear implantation.

### Study exclusion criteria

Studies including only either pre- or post-operative testing were excluded. Non-English language papers were excluded as their exclusion has been shown to have limited impact on review results [15]. Publications prior to 1980 were intentionally excluded.

### Types of outcome measures

1. Primary outcomes measures: pre and post operative vestibular function testing including at least one of: VEMPs, calorics, HIT, dynamic posturography.
2. Secondary outcome measures: subjective symptoms of vestibular function pre- and post-cochlear implantation.

### Information sources

A structured search was performed of the following three databases between 1980 and August 2017: Medline (Ovid), EmBase (Ovid), and PubMed. The reference list of each eligible study was also examined for extraction of relevant published articles not identified during the original search. A search of the grey literature, including unpublished data and PhD theses was also performed.

### Search strategy

The keywords, MeSH terms, and phrases searched included the following: “cochlear implant”, “pediatric”, “child” “VEMP”, “dizziness”, “posturography”, “vertigo” and “vestibular”. The search strategy was devised collectively and executed independently by two of the study authors (EY; MY). No study design or data limits were imposed upon the search. The full search strategy can be found in Appendix.

### Study selection and data collection process

The search results were collated on a Microsoft Excel (Microsoft, 2013) spreadsheet. The data collection sheet was piloted on two randomly-selected included studies and refined accordingly. Duplicate articles were eliminated. All abstracts were reviewed independently by authors EY and MY for relevance to the study topic. Full text studies of articles deemed to be relevant were further reviewed. Discrepancies regarding the inclusion or exclusion of studies were resolved through discussion between the two aforementioned authors. Neither of the review authors was blinded to the journal titles or the study authors or institutions.

### Data items/outcomes

The selected articles were searched for vestibular outcomes before and following cochlear implantation. VEMP testing, calorics, HIT, and posturography were the primary parameters. Demographic data on individual participants in each of the selected studies were gathered. These included, whenever available, etiology of hearing loss, imaging status, age at implantation, unilateral or bilateral implantation, method of implantation, simultaneous or sequential implantation, and timing of pre- and post-operative vestibular testing. Documentation of subjective post-operative vestibular symptoms was also extracted when possible. The two aforementioned authors independently extracted data from the selected articles according to these predetermined parameters. Any discrepancies in extracted data were resolved by discussion between the review authors.

### Risk of bias in individual studies

A checklist including ten key questions adapted from the Critical Appraisal Skills Programme by the Institute of Health at Oxford University was used for the risk of bias assessment [16]. Studies were categorized into one of three categories; (A) Low risk of bias; (B) Medium risk of bias; (C) High risk of bias. Both authors independently examined each study for risk of bias and assigned a categorization. Any discrepancies were resolved through discussion.

An assessment of the risk of meta-bias was also performed. This involved an independent review by both authors focusing on possible selective reporting within studies and a consideration of publication bias.

### Summary measures

The primary targeted measure calculated from the data was the relative risk of cochlear implantation on vestibular dysfunction. This was performed by comparing each child’s objective vestibular function pre-operatively to his or her vestibular function post-operatively. Abnormal test results were determined using each study authors’ definition.

### Planned methods of analysis

Separate analyses were performed on each individual study, and then a pooled analysis was performed on all studies to yield an overall relative risk. This involved a calculation of relative risk values, as well as I^2^ calculations and 95% confidence intervals. Statistical significance was defined as *p* < 0.05.

### Strength of evidence

The strength of evidence was determined by the significance of the meta-analysis, relevance of objective testing in the context of clinical impact, and the risk of bias assessment performed on all studies.

## Results

### Study selection

**Fig. 1:**
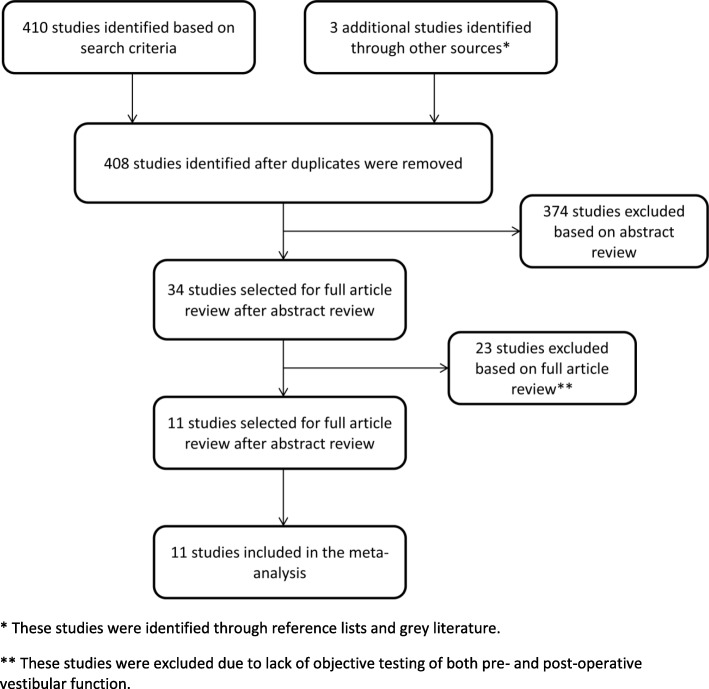
Study article inclusion/exclusion algorithm

Applying the predefined criteria, 410 studies were identified based on initial search of the databases (Fig. 1). An additional three studies were identified based on a review of study references and the grey literature. After duplicates were removed, a review of 408 abstracts resulted in exclusion of 374 studies that did not meet one or more of the inclusion criteria. Of the remaining 34 studies that underwent full text review, 23 were excluded based on participants over the age of 18 or not reporting both pre-operative and post-operative vestibular testing. The remaining 11 studies were included for full analysis.

### Study characteristics

Demographic data were extracted from all 11 studies which included study design, number of patients (study size), mean age, timing of pre-operative and post-operative vestibular testing, unilateral or bilateral implantation, sequential or simultaneous implantation, etiology of hearing loss, relevant imaging findings, cochleostomy site, and implant brand/model (Table 1) [18–28].

**Table 1:** Study demographics

Study	Design	Sample size	Mean age	Follow up	Side	Order	Etiology	Imaging malformation	Surgical site	Brand
Ajalloueyan	Prospective	27	2	6–8 weeks	Unilateral	N/A	Not specified	Not specified	Round Window	“Advanced Bionics Cochlear” and “Cochlear Nucleus”
Jin	Not specified	12	3.8	Not specified	Not specified	N/A	Not specified	Mondini (2), one branch of vestibulocochlear nerve (1), EVA (1)	Not specified	Not specified
De Kegel	Prospective	23	7mo	2 years	Both	Both	Genetic no Syndrome (4), Genetic syndrome (6), Acquired CMV/Meningitis/premature (8), ANSD (3), Unknown (2)	Not specified	Not specified	Not specified
Licameli	Prospective	19	8	4–6 weeks	Unilateral	N/A	Not specified	Not specified	Not specified	Not specified
Devroede	Retrospective	24	6.75	90 days post-second implant	Bilateral	Sequential	Syndrome (7), Genetic mutation (7), Meningitis (1), CMV (1), ANSD (2), Unknown (8)	Vestibular (3), cochlear (1), cochleo-vestibular (3)	Antero-inferior	“Cochlear”
Psillas	Prospective	10	2.85	6 months	Unilateral	N/A	Not specified	Not specified	Antero-inferior	“Freedom” (6) and “Medel Sonata” (4)
Xu	Prospective	31	5.5	4 weeks	Unilateral	N/A	Not specified	Not specified	Not specified	Not specified
Thierry	Retrospective	12	1.9	1.4 years	Unilateral	N/A	Genetic (8), Syndrome (7), CMV/meningitis (5), Inner ear / PDS mutation (7), Kallman (1), Unknown (15)	Bilateral malformation (7)	Antero-inferior	“Cochlear” and “Advanced Bionics”
Bogle	Retrospective	5	7.73	Not specified	Unilateral	N/A	Congenital (4), Acquired (1)	No abnormalities	Not specified	Not specified
Gupta	Prospective	23	5.48	6 weeks	Unilateral	N/A	Not specified	No abnormalities	Anterior-inferior	“Med-El Pulsar”
Hazzaa	Prospective	40	5.7	1 month and 6 months	Unilateral	N/A	Genetic (21), Syndrome (2), Ototoxicity (2), Perinatal (1), Unkown (13)	Not specified	Not specified	Not specified

Data pertaining to pre- and post-operative VEMPs, calorics, HIT, posturography, and subjective symptoms were collected (Table 2). Of the 11 studies, four reported subjective dizziness data and only one reported HIT data. No studies reported posturography data.

**Table 2:** Study vestibular findings

Study	VEMP	Calorics	vHIT	Non-video HIT	Symptoms
Ajalloueyan	Pre: 7% (2) no cVEMP bilaterally. 19% (5) no cVEMP unilaterally.	Pre: 8% (1) dysfunction.	Not performed	Pre: 0% had dysfunction.	N/A
		Post: No change.		Post: No change.	
	Post: 20% (8) abnormal cVEMP.				
Jin	Pre: 8% (1) reduced cVEMP, 42% (5) no cVEMP.	Not performed	Not performed	Not performed	N/A
	Post: 100% (12) abnormal cVEMP.				
De Kegel	Pre: 26% (5) had abnormal cVEMP unilaterally or bilaterally.	Not performed	Not performed	Not performed	Significant drop in gross motor performance, recovering toward age 2.
	Post: No change.				
Licameli	Pre: 12% (2) no cVEMP.	Not performed	Not performed	Not performed	Data not reliable collected, but no significant difference found.
	Post: 84% (16) abnormal cVEMP.				
Devroede	Pre: 21% (5) abnormal cVEMP.	Pre: 71% (17) abnormal calorics.	Not performed	Not performed	Post-op dizziness in 33% (3) who had post-op vestiublar dysfunction, which subsequently recovered.
	Post: 38% (9) abnormal cVEMP.	Post: 67% (16) abnormal calorics.			
Psillas	Pre: 30% (3) no cVEMP, 30% (3) reduced cVEMP.	Not performed	Not performed	Not performed	No dizziness, vertigo, instability, or nystagmus noted post-op.
	Post: 100% (10) abnormal VEMP.				
Xu	Pre: 26% (6) abnormal cVEMP.	Not performed	Not performed	Not performed	N/A
	Post: 65% (15) abnormal VEMP.				
Bogle	Pre: 0%abnormal cVEMP.	Not performed	Not performed	Not performed	N/A
	Post: 20% (1) abnormal cVEMP.				
Gupta	Not performed	Pre: 22% (5) abnormal calorics.	Not performed	Not performed	N/A
		Post: 35% (8) abnormal calorics.			
Hazzaa	Pre: 55% (22) abnormal or absent cVEMP.	Not performed	Not performed	Not performed	N/A
	Post: 80% (32) abnormal or absent cVEMP at 6 months.				

### Risk of bias within studies

A formal assessment of the risk of bias was performed on each study (Table 3). After review, 5 studies were considered to have a low risk of bias, 4 had a medium risk of bias, and 2 had a high risk of bias. All studies that were examined were either prospective or retrospective cohort studies.

**Table 3:** Risk of bias assessment

Study	Focused issue	Recruitment	Exposure accurately measured	Outcome accurately measured	Confounding factors	Follow up	Results	Precision of results	Believability of results	Applicability	Fit with evidence	Implications	Risk of bias
Ajalloueyan	Yes	Good	Yes	Yes	Steps taken to mitigate, limitations not formally addressed	Well documented	Well documented	Poor	Fair	Good	Unsure	Fair	B
Jin	Yes	Poor	Yes	Yes	Few steps taken to mitigate, limitations weakly addressed	Poorly described	Well documented	Fair	Fair	Good	Unsure	Unsure	C
De Kegel	Yes	Good	Yes	Yes	Steps taken to mitigate, limitations formally addressed	Well documented	Well documented	Fair	Good	Good	Unsure	Fair	A
Licameli	Yes	Good	Yes	Yes	Some steps taken to mitigate, limitations formally addressed	Well documented	Well documented	Fair	Good	Good	Good	Fair	A
Devroede	Yes	Fair	Yes	Yes	Steps taken to mitigate, limitations formally addressed	Well documented	Well documented	Fair	Fair	Good	Unsure	Fair	B
Psillas	Yes	Fair	Yes	Yes	Few steps taken to mitigate, limitations somewhat addressed	Well documented	Well documented	Fair	Fair	Good	Good	Unsure	B
Xu	Yes	Good	Yes	Yes	Some steps taken to mitigate, limitations somewhat addressed	Well documented	Well documented	Fair	Good	Good	Good	Fair	A
Thierry	Yes	Good	Yes	Yes	Steps taken to mitigate, limitations somewhat addressed	Well documented	Well documented	Fair	Good	Good	Good	Fair	A
Bogle	Yes	Poorly described	Yes	Poorly described	Not mitigated, limitations formally addressed	Poorly described	Poorly described, small sample size	Poor	Poorly described	Poor	Unsure	Poor	C
Gupta	Yes	Good	Yes	Yes	Some steps taken to mitigate, limitations not formally addressed	Well documented	Well documented	Fair	Fair	Good	Good	Fair	B
Hazzaa	Yes	Good	Yes	Yes	Some steps taken to mitigate, limitations not formally addressed	Well documented	Well described	Good	Good	Good	Good	Fair	A

### Results of individual studies

**Fig. 2:**
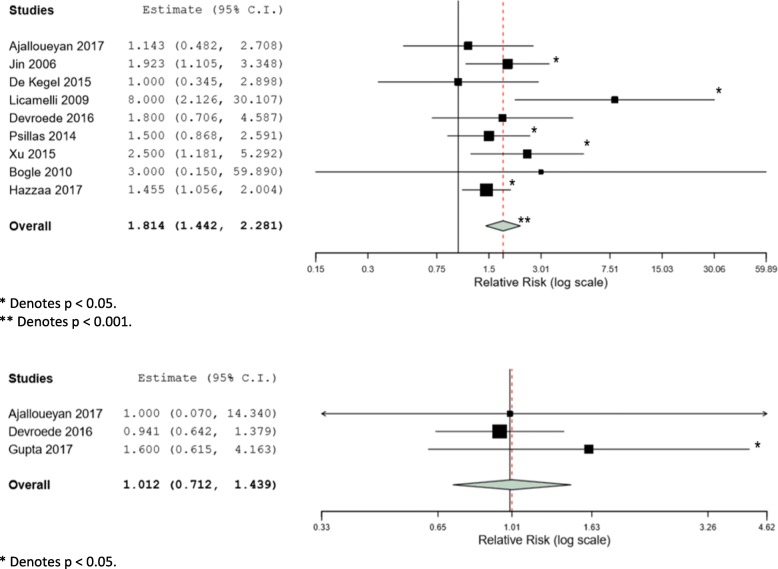
Meta-analysis Forest plots for VEMPs and caloric testing. 95% confidence intervals are included in parentheses. *Denotes a statistically significant result at the *p* < 0.05 level. ** Denotes a statistically significant result at the *p* < 0.001 level

Data extracted from individual studies were analyzed and relative risk values were calculated for each study. The results for the VEMP analyses are displayed in the Forest plot in Fig. 2. Five of the nine studies [18, 19, 21, 24, 28] that measured cervical VEMP (cVEMP) responses showed a statistically significant increase in abnormal VEMP responses following cochlear implantation. The pooled relative risk of 1.800 was supported by a 95% confidence interval that did not span 1 (95%CI 1.442–2.281) and a significant *p* value of less than 0.001. On review of all contributing studies, a VEMP change was defined as any significant change in threshold, amplitude, or latency measured post-operatively when that ear had baseline normal VEMP testing. A change was also defined as a complete loss of VEMP responses in an ear that had baseline abnormal VEMP testing (threshold, amplitude, or latency). These results represented Level 3 evidence according to OCEBM [17].

The results for the calorics analysis are displayed in the Forest plot in Fig. 3. One of the three studies [27] that measured calorics showed a statistically significant reduction in caloric responses following implantation. However, the pooled relative risk of 1.012 indicated no overall significant change and was based on only three different studies with the 95% confidence interval limits widely spanning below and above 1 (95%CI 0.712–1.439). Additionally, each of the three studies had a different definition of what constituted a significant caloric response change. Ajalloueyan et al. did not report a cut-off value and performed calorics using cold air, Gupta et al. defined a difference of > 15% in monothermal testing as significant and performed calorics using warm air, and Devroede et al. defined a difference of > 20% in bithermal testing and > 27% for monthermal testing as significant and performed monothermal calorics using cold water. This was deemed to represent Level 3 evidence.

Only four studies [18, 19, 23, 30] reported on subjective symptoms, and there were no studies that reported outcomes in a quantitative manner. Based on the qualitative reporting of data, no studies indicated that there were any post-operative symptoms such as imbalance or dizziness either immediately or long-term post-operatively.

### Synthesis of results

Data were combined for the purpose of a pooled meta-analysis. Relative risk, I^2^ value, and the 95% confidence interval were yielded and displayed together in the Forest plot (Fig. 2). The results showed a statistically significant increase in abnormal VEMP responses after either unilateral or bilateral cochlear implantation (RR 1.8, *p* < 0.001, 95%CI 1.57–2.02). The heterogeneity in the studies included for analysis was significant (I^2^ 91.86, Q = 98.304). A statistically significant increase in abnormal caloric testing was not similarly seen (*p* = 0.60).

### Risk of bias across studies

The risk of bias when comparing between studies was deemed to be low. There were no concerns identified over selective reporting of data, as patients in the studies that were reviewed were generally all accounted for in the results.

## Discussion

### Summary of evidence

Cochlear implantation carries a risk of post-operative vestibular dysfunction. Although its clinical significance currently appears to be minimal, long-term findings in the timeframe of decades have yet to be reported. Data from adult studies suggests that following implant surgery, otolithic organs and canal function can be compromised, leading to potential clinical manifestations such as post-operative imbalance, vertigo, and falls [1, 4, 7]. This is especially concerning for children who are more commonly undergoing bilateral implantation, raising the theoretical concern of bilateral vestibular dysfunction in the long term following surgical intervention. However, recent work on perceptual visual tilt using the static subjective visual vertical test has also shown that electrical pulses from the implant itself after initial cochlear implantation may have a role in actually improving vestibular function by helping to correct abnormal perception of vertical [29].

Cochlear implantation can potentially lead to disruption of saccular function in pediatric patients. Review of the literature would suggest there is a significant risk for increase in abnormal VEMP responses in pediatric patients undergoing cochlear implantation, with a relative risk of 1.8 (*p* < 0.001) despite several individual studies that did not display significant results [19, 20, 22, 23, 26]. These findings support similar findings published in a recent systematic review studying vestibular outcomes after adult cochlear implantation, showing a log relative risk of 0.5099 (*p* < 0.0001) after an analysis of 12 separate studies published since 2008 [9]. Proposed mechanisms for this finding have been previously discussed in the literature and include direct trauma related to insertion of the electrode, intraoperative perilymphatic fluid leakage, electrical stimulation of the otolithic organs, and foreign body labyrinthitis [17, 18].

There was no significant change in caloric responses in pediatric patients undergoing cochlear implantation. However, this is based on a small number of available studies reporting on this parameter and poor precision of available data. In addition, the caloric testing method and definition of canal dysfunction was widely variable, contributing to a significant source of bias.

There was a lack of available data concerning a number of the parameters of interest, specifically rotary chair, HIT and posturography. Challenges associated with collecting pediatric video-HIT data in particular have been reported in the past, citing difficulties such as lack of interest, involuntary or voluntary tensing of cervical muscles during testing, and problems with keeping eyes open during testing, especially in children under six years of age [30]. A significant proportion of pediatric implantations in this review occurred during the first five to six years of life.

Regardless of the objective results, there was no evidence for subjective symptoms of dizziness occurring in the pediatric population after cochlear implantation. Whether this is a limitation in available data or in the subjective reporting of symptoms, or reflective of a lack of clinically significant injury to the vestibular system in this population is uncertain.

### Limitations

There is a paucity of data on vestibular function in the pediatric population undergoing cochlear implantation; several studies were excluded because of a lack of pre-operative and post-operative objective measurements of vestibular function. As a result, there may be insufficient power to detect statistically significant differences in objective vestibular measures other than VEMPs. In addition, there was a general lack of data on motor function. Posturography can be difficult to perform on children and cannot be performed on young children and infants, which explains the lack of data in this category. Future studies examining vestibular function in implanted children may benefit from examining patients with scales such as the Ghent modification of the Alberta infant motor scale in order to provide age-standardized measures of motor function [30].

The amount of variance or heterogeneity observed among studies analyzing VEMP responses was substantial (I^2^ 91.86, Q = 98.304). Reasons could include inherent differences in the testing methods employed by different studies used to determine whether children displayed objective vestibular findings. Some studies reported detailed protocols of what constituted an abnormal VEMP response, including abnormalities in latency, amplitude, or threshold [20, 23, 24], whereas one study simply classified abnormal VEMP responses as hyporeflexic or areflexic [19]. Additionally, the mean time to follow-up was widely variable and ranged from as short as 4 weeks post-implant surgery to as long as two years post-operatively; vestibular dysfunction likely has the ability to recover or compensate over time.

Six of the eleven studies that were included in this review were graded as having a medium or high risk of bias due to poor documentation of follow-up, insufficient measures taken to reduce confounding factors in sample populations, and poor to fair precision of results.

The lack of reporting etiology of hearing loss and imaging findings in many of the studies may be relevant; certain conditions such as an enlarged vestibular aqueduct and other anomalous cochleovestibular anatomy have been shown to be responsible for higher rates of intraoperative CSF leaks, which could potentially put the patient at a higher risk for post-operative vestibular dysfunction [31].

We did not feel that there were significant limitations concerning reporting bias or incomplete retrieval of published research. The number of studies that were included in this analysis approximated the expected number based on similar studies on vestibular outcomes after cochlear implantation in adults [10].

Finally, cochlear implant electrode technology continues to improve and smaller diameter electrodes are facilitating atraumatic cochlear implantation. What role this may have in reducing the potential for vestibular injury in the future is uncertain.

### Conclusions

Pediatric patients experience a statistically significant change in vestibular function post-implantation as measured by VEMPs. This important finding supports previous work showing vestibular changes post-implantation in the adult population. However, data on post-operative subjective symptoms in the pediatric population is currently lacking. Additional longitudinal studies examining the correlation between VEMP changes and clinical symptoms may be warranted to determine the clinical significance of the findings in this review.

## Appendix A

### Full search strategy

The search on PubMed included:

((((cochlear implant [Title/Abstract]) OR cochlear implantation [Title/Abstract])) AND ((((paediatric [Title/Abstract]) OR pediatric [Title/Abstract]) OR child [Title/Abstract]) OR children [Title/Abstract])) AND ((((((((((vestibular [Title/Abstract]) OR VEMPs [Title/Abstract]) OR posturography [Title/Abstract]) OR dizziness handicap inventory [Title/Abstract]) OR DHI [Title/Abstract]) OR vertigo [Title/Abstract]) OR balance [Title/Abstract]) OR caloric [Title/Abstract]) OR vestibule [Title/Abstract]) OR labyrinth [Title/Abstract])

The search on Medline (Ovid) and Embase (Ovid) included:

((cochlear implant.mp. or exp. Cochlear Implants/) and (pediatric.mp. or exp. Pediatrics/) and (exp Dizziness/ or exp. Vestibule, Labyrinth/ or exp. Vertigo/ or exp. Vestibular Diseases/ or vestibular.mp.) and (exp Child/ or child.mp.))
